# A specimen-based database of small-eared shrews (Mammalia, Eulipotyphla, *Cryptotis*) in the Neotropical Region

**DOI:** 10.3897/BDJ.12.e135180

**Published:** 2024-10-22

**Authors:** Lázaro Guevara, Julieta Vargas-Cuenca, Yolanda Hortelano-Moncada, Fernando A. Cervantes

**Affiliations:** 1 Instituto de Biología, Universidad Nacional Autónoma de México, Mexico City, Mexico Instituto de Biología, Universidad Nacional Autónoma de México Mexico City Mexico; 2 Laboratorio Nacional Conahcyt de Biología del Cambio Climático, Mexico City, Mexico Laboratorio Nacional Conahcyt de Biología del Cambio Climático Mexico City Mexico

**Keywords:** Soricidae, Neotropics, vouchers, museum record, New World

## Abstract

**Background:**

This database compiles comprehensive occurrence information, based on voucher specimens of small-eared shrews, genus *Cryptotis*, that occur from México to Peru. The database integrates the information obtained from four main sources: natural history museums, public databases, fieldwork and scientific literature. It contains 3,639 records from 53 species in 12 countries. Of the total, 83.54% have collecting dates, 51.36% of the specimens are sexed and 84.56% have decimal degrees coordinates. By generating this database and making it publicly available, we hope to improve the biological knowledge of this group of small mammals still poorly studied in the region. It aims to be a valuable resource for students, researchers, conservationists and decision-makers.

**New information:**

The dataset contains information on all species of the genus *Cryptotis* in the Neotropical Region (namely from México to Peru), incorporating the most updated taxonomic and nomenclatural changes. The database includes records in regions and countries that are poorly represented in currently available data repositories. Most records have verified temporal and spatial information.

## Introduction

Species occurrence records publicly available in curated databases add value to primary biodiversity data by helping to evaluate the state of the art, determining knowledge gaps and guiding future research efforts (Erkimbaev et al. 2019, Swati et al. 2023). Given ongoing climate change, curated databases are particularly valuable for understanding biodiversity dynamics because they can provide evidence of range shifts associated with human activities (Tingley and Beissinger 2009). Unfortunately, information on the presence of species in digital repositories of taxa that are still poorly studied is often outdated, incomplete or even absent.

Shrews (Eulipotyphla, Soricidae) are small mammals playing an essential role in the structure and dynamics of populations of invertebrates, such as beetles, spiders and earthworms, on which they feed voraciously. These mammals are found in various habitats throughout most of the planet, but prefer humid tropical and temperate environments (Reumer 1995). Despite the high diversity and wide distribution of shrews, research on the natural history, taxonomy, systematics, distribution and ecology is still surprisingly patchy (Guevara et al. 2015,Amori et al. 2016, Noguera-Urbano et al. 2019). Recent large-scale reviews suggest that basic research on shrew species is urgently needed, particularly in tropical regions where they may be at serious risk of extinction due to combined pressures of land-use change and climate change (Amori et al. 2011, Kennerley et al. 2021).

Small-eared shrews of the genus *Cryptotis* are the third most diverse amongst the 29 genera that comprise the entire family (Database 2024). In less than 20 years, the number of species in the genus has increased from 30 to 54. This number is expected to grow due to increased taxonomic work on the group, fieldwork and the recent obtaining of genetic data (e.g. Guevara and Cervantes (2014), Woodman (2018), Zeballos et al. (2018)). The genus is distributed from southern Canada to northern South America, with a remarkable species increase towards Central México and Guatemala. In this region, the small-eared shrews have reached their greatest diversity and distribution, mainly restricted to mountain ecosystems, characterised by cold and moist forests. The growing research activity around the genus in the Neotropics has caused taxonomic and nomenclatural changes and increased information on species' presence in previously unexplored regions and on poorly-known taxa (Woodman et al. 2012, Lorenzo et al. 2019, Noguera-Urbano et al. 2019, Mejía-Fontecha et al. 2021, Guevara 2023).

During the last two decades, our working group has focused efforts on the study of systematics and biogeography of small-eared shrews in the Neotropical Region. We have integrated a curated database of 3,639 voucher specimens of the 53 small-eared shrews found in this region by combining previously published records on digital databases, new data from fieldwork, scientific literature and the revision of specimens in natural history museums (Suppl. material 1). This extensive database can be used for various purposes, including re-evaluating the taxonomy of species groups, conducting studies of intra- and interspecific variation, identifying areas with limited data, conducting ecological analyses using confirmed occurrence records and assessing conservation status. Developed to support specimen-based research, we hope this database will aid future work and enhance our knowledge of one of the least-studied mammal groups in this biodiversity hotspot.

## Sampling methods

### Study extent

**Figure 1.**
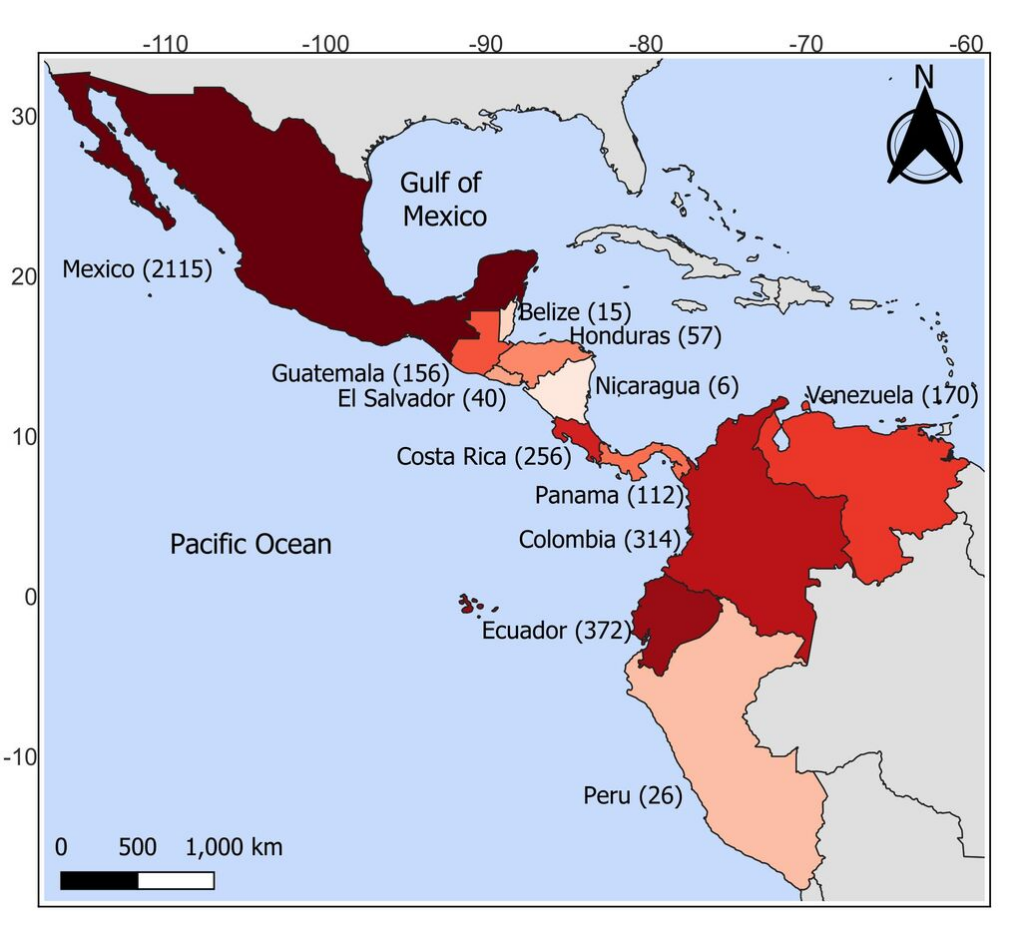
Records of small-eared shrews, *Cryptotis*, by country in the Neotropics. The intensity of the red colour indicates the greater number of specimens recorded.

The dataset contains records on 53 species of *Cryptotis* found in the territory from México to Peru (Fig. 1). The only species of the genus not included is *Cryptotisparvus*, which is of Nearctic affinity and distributed in the United States and Canada.

### Sampling description

Specimen records were obtained from four main sources: natural history museums, public online databases, literature and fieldwork.

**a)** Natural History Museums: We reviewed specimens in the following 21 scientific collections from Colombia, Ecuador, México and the United States: Colección Nacional de Mamíferos, Instituto de Biología, UNAM (CNMA; México City, México); Colección Mastozoológica, El Colegio de la Frontera Sur (ECO-SC-M; San Cristóbal de Las Casas, Chiapas); The University of Kansas, Natural History Museum (KU; Lawrence, Kansas); Museo de Zoología “Alfonso L. Herrera,” Facultad de Ciencias, UNAM (MZFC; México City, México); Colección Zoológica Regional Mammalia, Instituto de Historia Natural y Ecología (CZRMA; Tuxtla Gutiérrez, Chiapas); Museo de Zoología, Universidad de Ciencias y Artes de Chiapas (MZ-UNICACH; Tuxtla Gutiérrez, Chiapas); Colección Mastozoológica, CIIDIR-IPN (OAX-MA; Oaxaca de Juárez, Oaxaca); Colección de Mamíferos, CIByC, UAEM (CMC; Cuernavaca, Morelos); Colección de Mamíferos, Universidad Autónoma Metropolitana Unidad Iztapalapa (UAMI; México City, México); Colección de Mamíferos, Universidad Veracruzana (IIB-UV; Xalapa, Veracruz); National Museum of Natural History, Smithsonian Institution (USNM; Washington, D.C.); American Museum of Natural History (AMNH; New York, New York); Colección Teriológica de la Universidad de Antioquia (CTUA; Medellín Colombia); Instituto de Ciencias Naturales, Universidad Nacional (ICN; Bogotá, Colombia); Instituto de Investigación de Recursos Biológicos Alexander von Humboldt (IAvH, Villa de Leyva, Colombia); Pontificia Universidad Javeriana (MPUJ; Bogotá, Colombia); Museo de Historia Natural de la Universidad del Cauca (MHNUC, Popayán, Colombia); Museo de La Salle (MLS, Bogotá, Colombia); Universidad del Valle (UV; Cali, Colombia); Museo de Zoología de la Pontificia Universidad Católica del Ecuador (QCAZ; Quito, Ecuador); and Museo Ecuatoriano de Ciencias Naturales (MECN, Quito, Ecuador). The fluid bodies, skins, skulls and/or postcranial skeletons were reviewed to corroborate or determine the taxonomic identity and obtain or verify the collecting data and sex of specimens.

**b) Public online databases**: We consulted data in public databases of the following scientific collections that house specimens of shrews from the Neotropical Region: American Museum of Natural History (AMNH; https://emu-prod.amnh.org/db/emuwebamnh/Query.php); National Museum of Natural History, Smithsonian Institution (USNM; https://collections.nmnh.si.edu/search/mammals/), The University of Kansas, Natural History Museum (KU; https://biodiversity.ku.edu/mammalogy/collection-search), British Museum of Natural History (BMNH; https://doi.org/10.5519/qd.f94zgtmw); Instituto de Investigación de Recursos Biológicos Alexander von Humboldt (IAvH-M; http://i2d.humboldt.org.co/ceiba/resource.do?r=mamiferos_iavh). We also consulted the Arctos online collection management information system (https://arctos.database.museum/#), the database related to the project “Modelado de la distribución potencial de las musarañas (Mammalia, Soricidae)” from the Comisión Nacional para el Conocimiento y Uso de la Biodiversidad (http://www.conabio.gob.mx/institucion/cgi-bin/datos2.cgi?Letras=JM&Numero=44) and the DataWebEcuador, a portal to access data of specimens from the collections of Ecuador (https://bioweb.bio/portal/). We also compare the information with what is available in the Global Biodiversity Information Facility through a download on 8 April 2024 (https://doi.org/10.15468/dl.qtdnkn).

**c) Literature**: Due to the intense study of *Cryptotis* in the last 20 years, we carefully reviewed the most recent literature to incorporate taxonomic and nomenclatural changes to the database. Additionally, we include new records that have not yet been digitised or are not still available in any database (Suppl. material 2).

**d) Fieldwork**: Since 2003, we have carried out fieldwork aimed at the scientific collecting of shrews in nine States of México: Chiapas, Colima, Estado de México, Hidalgo, Oaxaca, Puebla, San Luis Potosí, Tamaulipas and Veracruz. For catching shrews, we combined Sherman traps and pitfall traps. The localities were georeferenced in the field using global positioning systems. All scientific collecting was conducted under permits issued by the SEMARNAT (FAUT 006) and prior authorisation from the local authorities. We followed standard recommendations on specimen capture, sacrifice and preparation of small mammals (Sikes et al. 2016). All shrews are deposited at the Colección Nacional de Mamíferos (CNMA).

### Quality control

Original records were curated to confirm species identification and verify temporal and spatial congruence. We eliminated duplicates and records in which it was not possible to find or verify the institution that houses the voucher specimen. The final species list and type status were checked according to the Mammal Diversity Database (Burgin et al. 2018,Database 2024) and Woodman (2018). Valid names were carefully reviewed and followed the International Code of Zoological Nomenclature. The collection date was separated into day, month and year, to facilitate temporal analysis. The locality for each record was also inspected to check its congruence with the distribution of the species according to the literature examined. The decimal degrees georeferenced of the records were verified or obtained using Google Earth Pro 7.3.6.9796 and following recommendations from Liao et al. (2022). Ambiguous localities or locations with insufficient information were kept without assigned georeferences.

## Geographic coverage

### Description

Neotropical countries, from México to Peru.

### Coordinates

-5.7001 and 27.1672 Latitude; -105.6843 and to -67.0834 Longitude.

## Taxonomic coverage

### Taxa included

**Table:** 

Rank	Scientific Name	Common Name
kingdom	Animalia	Animals
phylum	Chordata	Chordates
subphylum	Vertebrata	Vertebrates
class	Mammalia	Mammals
subclass	Theria	Therian mammals
order	Eulipotyphla	
family	Soricidae	Shrews
subfamily	Soricinae	
tribe	Blarinini	

## Temporal coverage

### Notes

**Figure 2.**
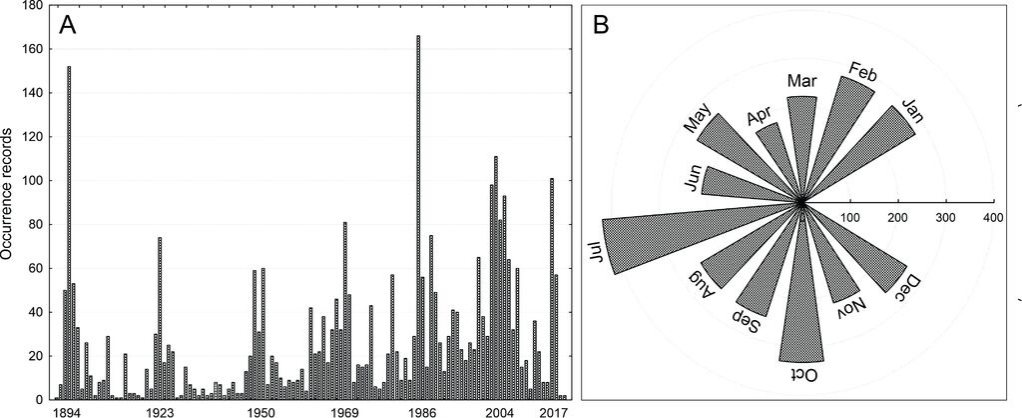
Records of small-eared shrews, *Cryptotis*, by year (A) and month (B) in the Neotropics.

Based on dates obtained from multiple sources, this database contains data throughout the year and from 1891 until the beginning of the COVID-19 pandemic. However, it is prudent to mention that some records were undoubtedly obtained before 1891, although the date is imprecise and remains undetermined (Fig. 2).

## Usage licence

### Usage licence

Open Data Commons Attribution License

## Data resources

### Data package title

A specimen-based database of small-eared shrews (Mammalia, Eulipotyphla, *Cryptotis*) in the Neotropical Region

### Resource link

https://www.gbif.org/dataset/004d2940-48cc-4d6f-9b49-59c6b5dac421

### Number of data sets

1

### Data set 1.

#### Data set name

Small-eared shrews (Mammalia, Cryptotis) in the Neotropical Region v.1.1.

#### Data format

Darwin Core format

#### Download URL

https://www.gbif.org/dataset/004d2940-48cc-4d6f-9b49-59c6b5dac421

#### Description

This database (Suppl. material 1) compiles comprehensive occurrence information, based on voucher specimens of small-eared shrews, genus *Cryptotis*, that occur from México to Perú. The database integrates the information collected from four main sources: natural history museums, public databases, fieldwork and scientific literature. It contains 3,678 records from 53 species in 12 countries. This database is available through the Global Biodiversity Information Facility.

**Data set 1.** 

Column label	Column description
occurrenceID	The unique identifier of the record constructed with the essential information of the voucher specimen (i.e. collectionCode and catalogNumber).
basisOfRecord	The specific nature of the data record. As this database is about voucher specimens, then they all have the value PreservedSpecimen.
ownerInstitutionCode	The name (or acronym) in use by the institution having ownership of the specimen.
collectionCode	The acronym identifying the collection from which the record was derived.
catalogNumber	Unique identifier for the record within the dataset or collection.
scientificName	The full scientific name, with authorship and date information.
acceptedNameUsage	The full name of the currently valid taxon.
scientificNameAuthorship	The authorship information for the scientific name according to the conventions of the ICZN.
previousIdentifications	A list of previous assignments of names to the specimen.
typeStatus	A list of nomenclatural types applied to the specimen.
kingdom	The full scientific name of the kingdom in which the taxon is classified.
phylum	The full scientific name of the phylum or division in which the taxon is classified.
class	The full scientific name of the class in which the taxon is classified.
order	The full scientific name of the order in which the taxon is classified.
family	The full scientific name of the family in which the taxon is classified.
genus	The full scientific name of the genus in which the taxon is classified.
specificEpithet	The name of the first or species epithet of the scientific name.
infraspecificEpithet	The name of the lowest or terminal infraspecific epithet.
taxonRank	The taxonomic rank of the most specific name.
eventDate	The date-time during which the record occurred.
year	The four-digit year in which the record occurred.
month	The integer month in which the record occurred.
day	The integer day in which the record occurred.
countryCode	The standard code for the country in which the locality occurs.
country	The name of the country in which the location occurs.
stateProvince	The name of the next smaller administrative region than country.
county	The full, unabbreviated name of the next smaller administrative region than state or province.
locality	The specific description of the place.
decimalLatitude	The geographic latitude in decimal degrees.
decimalLongitude	The geographic longitude in decimal degrees.
geodeticDatum	The ellipsoid, geodetic datum or spatial reference system (SRS), upon which the geographic coordinates are given in decimal latitude and decimal longitude.
individualCount	The number of individuals present at the time of the occurrence.
recordedBy	A list of names of people, groups or organisations responsible for recording the specimen.
sex	The sex of the individual.
preparations	A list of preparations and preservation methods for the specimen.
taxonRemarks	Species group to which the taxon belongs according to Woodman (2019).

## Supplementary Material

7810C0B6-3686-59D5-975B-3510AEDD4809 10.3897/BDJ.12.e135180.suppl1 Supplementary material 1 A specimen-based database of small-eared shrews (Mammalia, Eulipotyphla, Cryptotis) in the Neotropical Region Data type A database CSV Brief description This database compiles comprehensive occurrence information, based on voucher specimens of small-eared shrews, genus *Cryptotis*, that occur from México to Perú. The database integrates the information collected from four main sources: natural history museums, public databases, fieldwork and scientific literature. It contains 3,639 records from 53 species in 12 countries. File: oo_1144456.csv https://binary.pensoft.net/file/1144456 Lázaro Guevara, Julieta Vargas-Cuenca, Yolanda Hortelano-Moncada, Fernando A. Cervantes

C80296C9-203D-516C-9C8F-F66F152AA08D 10.3897/BDJ.12.e135180.suppl2 Supplementary material 2 Scientific literature consulted to incorporate new records and recent taxonomic and nomenclatural changes of the genus Cryptotis Data type Table Brief description A table describing the consulted scientific literature to incorporate new records and recent taxonomic and nomenclatural changes of the genus *Cryptotis*. File: oo_1115600.csv https://binary.pensoft.net/file/1115600 Lázaro Guevara, Julieta Vargas-Cuenca, Yolanda Hortelano-Moncada, Fernando A. Cervantes
